# White matter injury after neonatal encephalopathy is associated with thalamic metabolite perturbations

**DOI:** 10.1016/j.ebiom.2020.102663

**Published:** 2020-02-12

**Authors:** Paolo Montaldo, Phoebe Ivain, Pete Lally, Paul Bassett, Stuti Pant, Vania Oliveira, Josephine Mendoza, Maria Morales, Ravi Swamy, Seetha Shankaran, Sudhin Thayyil

**Affiliations:** a Centre for Perinatal Neuroscience, Department of Brain Sciences, Imperial College London, London, UK; b Department of Neonatal Intensive Care, Università degli Studi della Campania Luigi Vanvitelli, Naples, Italy; c Statsconsultancy Ltd., Amersham, UK; d Neonatal-Perinatal Medicine, Wayne State University, USA

**Keywords:** Therapeutic hypothermia, Magnetic resonance spectroscopy, Magnetic resonance imaging, Biomarkers, Neonatal encephalopathy

## Abstract

**Background:**

Although thalamic magnetic resonance (MR) spectroscopy (MRS) accurately predicts adverse outcomes after neonatal encephalopathy, its utility in infants without MR visible deep brain nuclei injury is not known. We examined thalamic MRS metabolite perturbations in encephalopathic infants with white matter (WM) injury with or without cortical injury and its associations with adverse outcomes.

**Methods:**

We performed a subgroup analysis of all infants recruited to the MARBLE study with isolated WM or mixed WM/cortical injury, but no visible injury to the basal ganglia/thalamus (BGT) or posterior limb of the internal capsule (PLIC). We used binary logistic regression to examine the association of MRS biomarkers with three outcomes (i) WM injury score (1 vs. 2/3); (ii) cortical injury scores (0/1 vs. 2/3); and (iii) adverse outcomes (defined as death, moderate/severe disability) at two years (yes/no). We also assessed the accuracy of MRS for predicting adverse outcome.

**Findings:**

Of the 107 infants included in the analysis, five had adverse outcome. Reduced thalamic N-acetylaspartate concentration [NAA] (odds ratio 0.4 (95% CI 0.18–0.93)) and elevated thalamic Lactate/NAA peak area ratio (odds ratio 3.37 (95% CI 1.45–7.82)) were significantly associated with higher WM injury scores, but not with cortical injury. Thalamic [NAA] (≤5.6 mmol/kg/wet weight) had the best accuracy for predicting adverse outcomes (sensitivity 1.00 (95% CI 0.16–1.00); specificity 0.95 (95% CI 0.84–0.99)).

**Interpretation:**

Thalamic NAA is reduced in encephalopathic infants without MR visible deep brain nuclei injury and may be a useful predictor of adverse outcomes.

**Funding:**

The National Institute for Health Research (NIHR).

**Research in context**

***Evidence before this study***

**Relevant studies were identified through Pubmed, Embase and Web of Knowledge in the time period of 2005 to 2018 by utilising the following keywords: [Magnetic resonance imaging OR diffusion-weighted imaging OR magnetic resonance spectroscopy OR fractional anisotropy OR diffusion tensor] AND [hypoxic ischaemic encephalopathy or neonatal encephalopathy] AND [newborn OR neurodevelopmental outcome] AND [Therapeutic Hypothermia]. A total of 620 articles were collected of which 45 full-text papers were obtained. Of these, 22 papers met eligibility, with 11 reporting the number of patients with visible basal ganglia/thalamic injury (BGT) as determined by conventional magnetic resonance imaging (MRI). BGT injury (median) was reported in 30.1% (range 6%−60%) of the cooled infants with neonatal encephalopathy. There were no studies examining the utility of BGT MR spectroscopy in infants without visible BGT injury on conventional MRI or in isolated white matter injury.**

***Added value of this study***

**Thalamic [NAA] is reduced in encephalopathic infants with white matter injury and is an accurate predictor of later adverse outcomes, irrespective of the pattern of brain injury. Therefore, thalamic [NAA] may be a useful surrogate outcome measure for early phase neuroprotection trials.**

***Implications of all the available evidence***

**The implementation of [NAA] as a surrogate marker of outcome would not only improve future multi-centre neuroprotection trials but contribute to our understanding of the underlying pathophysiology of injury occurring in the neonatal brain following neonatal encephalopathy.**

Alt-text: Unlabelled box

## 1 Introduction

Neonatal encephalopathy (NE) is the leading identifiable cause of long-term neurological disability affecting 1–2 infants per 1000 live births in high-income countries [1]. Over the past decade, major trials have provided definitive evidence regarding the efficacy and safety of therapeutic hypothermia in cases of moderate and severe encephalopathy in high-income-countries. This is now considered the standard of care for NE [1].

Preclinical models have demonstrated that neuronal death after a hypoxic-ischaemic insult is the consequence of impaired neuronal metabolism, particularly in brain regions with high metabolic activity, such as the basal ganglia, thalami and hippocampus structures [2,3]. Basal ganglia/thalami (BGT) and cortical injury have been classically linked to acute hypoxic ischaemia. Grey matter neurons and early myelinating tissues within the immature brain have a higher metabolic rate than the surrounding white matter (WM) and are significantly more vulnerable to acute anoxia. White matter injury (WMI) instead, has been traditionally linked to sub-acute or chronic hypoxic ischaemia [4].

Despite the substantiated histological evidence for BGT injury after an acute hypoxic ischaemic insult in preclinical models, about 6–60% of the cooled encephalopathic infants exhibit BGT injury when using conventional magnetic resonance imaging (MRI) [5], [6], [7], [8], [9], [10], [11]. This variation may reflect the difficulty in identifying subtle BGT damage. While MRI is a useful tool for assessing the macroscopic brain injury after NE, it does not identify metabolic perturbations [12].

There is a shortage of evidence regarding the relationship between structural MRI and magnetic resonance spectroscopy (MRS) biomarkers, which would permit further insight as to the connection between macroscopic brain injury and cerebral metabolic perturbation. WMI also occurs in cooled infants with NE [8,13] and therefore, understanding the underlying pathophysiological processes of WMI is important for the validation of surrogate MR biomarkers [14].

The first aim of this study was to explore the relationship between thalamic MRS biomarkers and the degree of WM and cortical MRI injury scores in a group of infants devoid of visible damage to the BGT and the posterior limb of internal capsule (PLIC). The second aim of this study was to assess the prognostic accuracy of the thalamic MRS biomarkers in these neonates.

We hypothesised that in the absence of MR visible injury to the BGT/PLIC, thalamic [NAA] is a more sensitive marker of injury compared to conventional MRI in infants with metabolite perturbation on MRS.

## 2 Methods

We conducted a secondary analysis of the Magnetic Resonance Biomarkers in Neonatal Encephalopathy (MARBLE, NCT01309711) study, a prospective observational study, which involved 8 different neonatal intensive care units across the UK and USA. A total of 223 term babies with evidence of NE and undergoing therapeutic hypothermia were recruited between January 2013 and June 2016 [11,15].

All parents gave written informed consent and the study was approved by the North London Research Ethics Committee (13HH1843) and each of the participating clinical sites [15].

The secondary analysis reported in this study included 107 infants with isolated WM or mixed WM/cortical injury (defined as WM > 0), who lacked any visible injury to the BGT or PLIC (BGT, PLIC = 0). MRI visible injury was assessed using a validated scoring system [8]. Brain tissue injury was assessed from 0 to 3 for BGT(0 = normal, 1 = mild (focal abnormal signal intensity), 2 = moderate (multifocal abnormal signal intensity), 3 = severe (widespread abnormal signal intensity), WM (0= normal, 1 = mild signal abnormalities with long T1 and T2 in periventricular WM only, 2 = moderate signal abnormality with long T1 and T2 extending to the subcortical WM and/or focal punctate lesions or focal area of infarction, and 3=severe widespread abnormalities with overt infarction, haemorrhage, and long T1 and T2) and cortex (0 = normal, 1 = mild signal abnormality with 1–2 sites involved, 2 = moderate signal abnormality with 3 sites involved, 3=severe signal abnormality involving more than 3 sites). The PLIC was scored from 0 to 2 (0 = normal, 1 = equivocal, 2 = abnormal). The presence of focal hemispheric lesions and their site were also noted [8].

We performed MR imaging between 4 and 14 days after birth (i.e. after completion of therapeutic hypothermia) in all infants using a 3 Tesla MRI scanner (Philips, Siemens or GE). The MR sequences were harmonised for use across all sites [15]. A paediatric neurologist (ST) with 10 years of MR experience reported the MR images while masked to the clinical and spectroscopy data. Based on the MRS protocol previously described [11], all water suppressed spectra were analysed using LCModel (v6.3–1 J), with basis sets simulated using VeSPA (v0.9.11). The methyl peaks of NAA, NAAG, choline (Cho), phosphocreatine (PCr), and creatine (Cr) were identified and separated from other groups in the basis spectra so as to quantify individual relaxation rates. NAA+NAAG methyl peaks at ~2.0 ppm were combined and referred to as ‘NAA’, PCr + Cr methyl peaks at ~3.0 ppm were combined and referred to as ‘Cr’, and Thr+Lac were combined and referred to as ‘Lac’ due to strong covariance. We used a single peak to fit the choline signal at ~3.2 ppm and referred to it as ‘Cho’. Water unsuppressed signals were quantified using HLSVD. The parenchymal water signal was quantified by biexponential fit of the water unsuppressed series, with a long T2 component (fixed at 500 ms) accounting for mobile water. NAA/Cho, NAA/Cr and Lac/NAA were all derived from the first acquisition (TR/TE = 2288/288 ms). [NAA] was calculated from the fitted NAA methyl singlets throughout the experiment with comparison of the relaxation corrected signal to the relaxation corrected unsuppressed water signal after partial volume correction [11]. The MRS postprocessing and analysis were performed centrally by a single MR physicist (PJL) masked to the clinical and conventional MRI data.

Upon recruitment at each site, the encephalopathy stage was assessed using a standardised and validated NICHD neurological examination by a certified examiner [11].

Neurodevelopmental outcome was determined at two years of age using the Bayley Scales of Infant and Toddler Development (BSID-III), with the examiner masked to the MRS information. Vision and audiometric evaluations were performed alongside the collection of anthropometric data (weight, height, and head circumference) as part of the follow-up assessments by trained personnel. The presence and type of cerebral palsy was determined according to Gorter et al. [16] and severity was graded using the five levels defined in the Gross Motor Function Classification System (GMFCS), from 1 (minimal impairment) to 5 (severe impairment with dependence on carers for most daily activities).

Disability was classified as severe if any one of the following criteria were met: Bayley III, both cognitive and language composite score <70; GMFCS level 3–5; hearing impairment requiring hearing aids; or blindness. Moderate disability was defined as both cognitive and language composite scores between 70–84 and one or more of the following: GMFCS level II; hearing impairment with no amplification; or a persistent seizure disorder [11]. We defined an adverse outcome as death or moderate/severe disability.

### 2.1 Data statement

Our data set will be accessible to all readers upon reasonable request and following the completion of our sub-studies.

### 2.2 Statistical analysis

There were three main study outcomes, each of them was either binary in nature or grouped into two categories. These were: (i) WMI (score 1 vs. 2/3); (ii) cortical injury (scores 0/1 vs. 2/3; and (iii) adverse outcome (defined as death, moderate or severe disability) (yes vs. no).

Binary logistic regression analysis was performed to determine whether the thalamic MRS biomarkers were associated with any of the three outcomes. Each biomarker was considered in a separate analysis. The analyses for WM score were first performed unadjusted and then adjusted for gestational age and age at time of MRI. Due to the small number of subjects in one of the outcome categories for adverse outcome and cortical score, only an unadjusted analysis was performed

Receiver operating characteristic curves were created to analyse the accuracy of each biomarker in predicting adverse outcomes. Cut points for each parameter were based on those used in the analysis of the whole MARBLE dataset. The cut-off values for each variable tested were: [NAA] (≤5.6 mmol/kg/wet weight, which corresponded to 80% of the mean [NAA] in patients with good outcome), Lactate/NAA (>0.22), NAA/Choline (≤0.85), NAA/Creatine (≤1.29), WM (>2), and cortex (>1) [11]. The sensitivity and specificity of the biomarkers in predicting adverse outcome with 95% confidence intervals (CIs) were obtained with the exact binomial method and represented as Forest plots.

## 3 Results

Of the total 223 cooled infants recruited to the MARBLE study, 58 (26%) had normal MRI, 52 (22%) had BGT/PLIC injury with or without periventricular or sub-cortical WMI, and 6 (3%) had isolated cortical injury and were excluded. The remaining 107 infants, who did not have any visible BGT or PLIC injury on conventional MRI, were included in the analysis. Of these, 85 (80%) had isolated WMI and 22 (20%) had combined WM and cortical injury. Ninety-two (86%) of these infants were assessed at two years of age and 15 (14%) were lost to follow-up. Of the 92 infants assessed at two years, five (5.4%) had adverse outcomes (four moderate disability, one severe disability, no deaths). The clinical characteristics of the overall cohort and those who were followed up at two years were found to be similar (Table 1).

**Table 1:** Clinical characteristics of the patient cohort.

	Total participants (*N* = 107)	Follow-up at 2 years (*N* = 92)
Gestational age (weeks)	39.7 (1.6)	39.7 (1.6)
Birth weight (kg)	3.37 (0.5)	3.37 (0.5)
Cord pH	6.9 (0.1)	6.9 (0.1)
Apgar 5 min	4 [3]	4 [3]
Seizures <6 h	46 (43%)	41 (44.6%)
Intrapartum events	16 (15%)	15 (16.3%)
Ruptured uterus	3 (2.8%)	2 (2.2%)
Cord prolapse	2 (1.9%)	2 (2.2%)
Shoulder dystocia	8 (7.5%)	8 (8.7%)
Obstructed labour	3 (2.8%)	3 (3.3%)
HIE Stage		
Mild	19 (18%)	17 (18.5%)
Moderate	81 (76%)	67 (72.8%)
Severe	7 (6%)	7 (7.6%)
Age at MRI (days)	8.8 (4.8)	8.6 (5.1)
Positive blood culture	2 (1.9%)	2 (2.2%)
Hypoglycaemia	22 (20.5%)	18 (19.6%)
Cerebral palsy	8 (7.5%)	8 (8.7%)
Prolonged Rupture of Membrane	20 (18.7%)	18 (19.6%)
Reduced foetal movements	15 (14%)	14 (15.2%)
Abnormal cardiotocogram	61 (57%)	78 (84.8%)
Bradycardia	22 (20.6%)	17 (18.5%)
Late decelerations	11 (9.3%)	6 (6.5%)
Other abnormality	17 (15.9%)	17 (18.5%)
Variable decelerations	9 (8.4%)	7 (7.6%)
Sinusoidal	2 (1.9%)	2 (2.2%)
Antepartum haemorrhage	8 (7.5%)	7 (7.6%)
Meconium stained liquor	41 (38.3%)	36 (39.1%)
Emergency LSCS	45 (42.1%)	39 (42.4%)
Resuscitation	103 (96.3%)	88 (95.7%)
Bag and mask/T Piece	32 (29.9%)	27 (29.3%)
Intubation	30 (28%)	26 (28.3%)
Cardiac Massage	23 (21.5%)	21 (22.8%)
Fluids	5 (4.7%)	3 (3.3%)
Drugs	2 (1.9%)	2 (2.2%)

*The data is represented by n (%), median [IQR] or mean (SD). Percentages are in terms of the number of mothers or neonates for whom data were available. LSCS=lower segment caesarean section*.

Of the five patients with adverse outcome, two had intrapartum sentinel events; one with ruptured uterus and the other with shoulder dystocia. There was no evidence of hypoglycaemia or positive blood cultures amongst these infants. Furthermore, two of the patients had seizures upon admission to the neonatal intensive care unit.

Within our cohort, [NAA] absolute quantification was completed in 49 infants (four were lost to follow-up, with 45 assessed at two years, of which one had moderate disability, one had severe disability, and none died). Lactate/NAA peak-area ratio was quantified in 90 patients (13 were lost to follow-up, with 77 assessed at two years, of which four had moderate disability, one had severe disability, and none died). The clinical features of patients with [NAA] and Lactate/NAA quantification are shown in Supplementary Table 4.

**Fig. 1:**
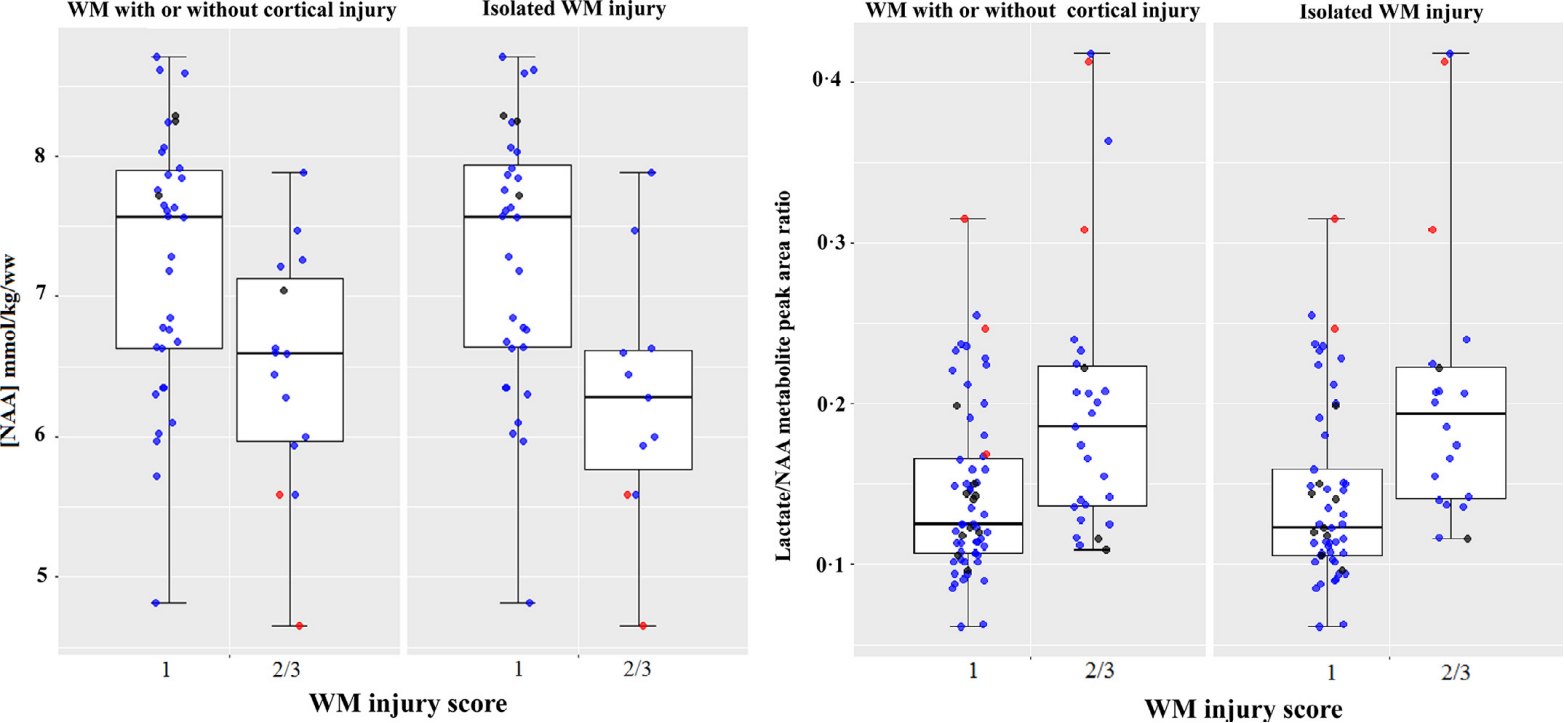
Boxplots of proton thalamic MRS [NAA] and Lactate/NAA values for neonates with WM injury with or without cortical injury compared to those with isolated MRI WM injury. The boxplots exhibit the spread of data points across the median and IQR for each injury score group. Medians are represented by horizontal lines; the boxes represent the upper and lower quartiles; and the whiskers indicate 1.5xIQR from upper and lower quartiles. Blue dots indicate normal outcome, red dots are patients with adverse outcomes, and the black dots are those with unknown outcomes.

Infants with more severe grades of WMI (grade 2/3) were shown to have lower [NAA] (median: 6.58, IQR: 1.28) and higher Lactate/NAA peak-area ratio (median: 0.18, IQR:0.09) compared to WMI=1 (NAA- median: 7.56, IQR: 1.38; Lactate/NAA- median: 0.12, IQR: 0.06). When we considered only patients with isolated WMI, [NAA] was significantly lower (median: 6.28, IQR: 0.92) and Lactate/NAA higher (median: 0.19, IQR: 0.08) in more severe cases (grade 2/3) compared to WMI=1 (NAA- median: 7.56, IQR: 1.37; Lactate/NAA- median: 0.12, IQR: 0.06) (Fig. 1).

The thalamic [NAA] and Lactate/NAA peak-area ratio were associated with WM MRI scores when using binary logistic regression adjusted for gestational age and age at time of MRI ([NAA]: odds ratio 0.4 (95% CI 0.18–0.93), *p* = 0.033; Lactate/NAA: odds ratio 3.37 (95% CI 1.45–7.82), *p* = 0.005). The [NAA] ≤5..6 mmol/kg/wet weight (80% of the mean [NAA] in patients with good outcome) model correctly identified 90.9% of patients with a WMI score of 1 and 33.3% of patients with a WMI score of 2 or 3, thus displaying an overall correct prediction rate of 72.9%. The Lactate/NAA >0.22 model correctly identified 90.2% of patients with a WMI score of 1 and 29.6% of patients with a WMI score of 2 or 3, with an overall prediction rate of 71.6%. NAA/Choline and NAA/Creatine did not show a significant association with WMI (Supplementary Table 1). Furthermore, none of the assessed MRS markers demonstrated a significant relationship with cortical injury (Supplementary Table 2).

Binary logistic regression also revealed a significant association between thalamic [NAA] and Lactate/NAA peak-area ratios with adverse outcome ([NAA]: *p* = 0.04; Lactate/NAA: *p* = 0.003). The odds ratio for [NAA] was 0.06 (95% CI 0.004–0.88) and Lactate/NAA was 5.97 (95% CI 1.86–19.14). The [NAA] model correctly identified 97.7% of the patients with normal outcome and 50% of those with adverse outcome, with an overall percentage of 95.6%. The Lactate/NAA model correctly identified 97.2% of the patients with normal outcome and 20% of those with adverse outcome, for an overall percentage of 92.2%. NAA/Choline and NAA/Creatine were not found to have a significant relationship with adverse outcome (Supplementary Table 3).

Thalamic [NAA] was found to have an AUC of 0.99 (95% CI 0.96–1.0), thus demonstrating the greatest predictive accuracy of the biomarkers assessed. [NAA] also exhibited the highest sensitivity: 1.00 (95% CI 0.16–1.00) and specificity 0.95 (95% CI 0.84–0.99) when using the cut-off value of ≤5.6 mmol/kg/wet weight (Table 2). The prognostic accuracy of thalamic [NAA] in infants with WMI was similar to the overall MARBLE cohort, which included all brain injury patterns (AUC: 0.99 (95% CI 0.94–1.00), sensitivity: 1.00 (95% CI 0.74–1.00), specificity: 0.97 (95% CI 0.90–1.00)) [11]. Hence indicating that the prognostic accuracy of NAA was independent of the pattern of brain injury.

**Table 2:** Accuracy of MRS and MRI biomarkers when prognosticating neurological outcome at two years. 95% CIs represented in parentheses. MRI scores for cortex were: 0=normal, 1=mild injury, 2=moderate injury. MRI scores for WM were: 0=normal, 1=mild injury, 2=moderate injury, 3=severe injury.

	Total patients	Patients lost to follow-up	Patients with adverse outcome	True positive: false positive	True negative: false negative	Sensitivity	Specificity	Positive likelihood ratio	Negative likelihood ratio	Area under the ROC curve
**MRI appearance**										
Cortex (score ≥1)	107	15	5 (5.4%)	1: 18	69: 4	0.20 [0.01–0.72]	0.79 [0.69–0.87]	0.97	1.01	0.52 [0.28–0.75]
White matter (≥2)	107	15	5 (5.4%)	2: 27	60: 3	0.40 [0.05–0.85]	0.69 [0.58–0.79]	1.29	0.42	0.54 [0.30–0.78]
**MR spectroscopy**										
NAA/Choline ratio (≤0.85)	90	13	5 (6.4%)	3: 37	35: 2	0.60 [0.15–0.95]	0.49 [0.37–0.61]	1.17	0.82	0.54 [0.30–0.78]
NAA/Creatine ratio (≤1.29)	90	13	5 (6.4%)	1: 9	63: 4	0.20 [0.01–0.72	0.88 [0.78–0.94]	1.60	0.91	0.61 [0.36–0.87]
Lactate/NAA ratio (>0.22)	90	13	5 (6.4%)	4: 12	60: 1	0.80 [0.28–1.00]	0.83 [0.73–0.91]	4.80	0.24	0.91 [0.80–1.00]
[NAA] (≤5.6 mmol/kg wet weight)	49	4	2 (4.4%)	2: 2	41: 0	1.00 [0.16–1.00]	0.95 [0.84–0.99]	21.5	0	0.99 [0.96–1.00]

In comparison, the prognostic accuracy of Lactate/NAA was lower (AUC: 0.91(95% CI 0.80–1.00), sensitivity: 0.80 (95% CI: 0.28–1.00), specificity: 0.83 (95% CI: 0.73–0.91)) in infants who were devoid of BGT injury compared to the overall MARBLE cohort (AUC: 0.94 (95% CI: 0.84–0.97), sensitivity: 0.88 (95% CI: 0.70–0.98), specificity: 0.90 (95% CI: 0.84–0.95)) [11]. Thus, thalamic Lactate/NAA may be an inferior biomarker to thalamic [NAA] in infants with mixed WM/cortical injury.

**Fig. 2:**
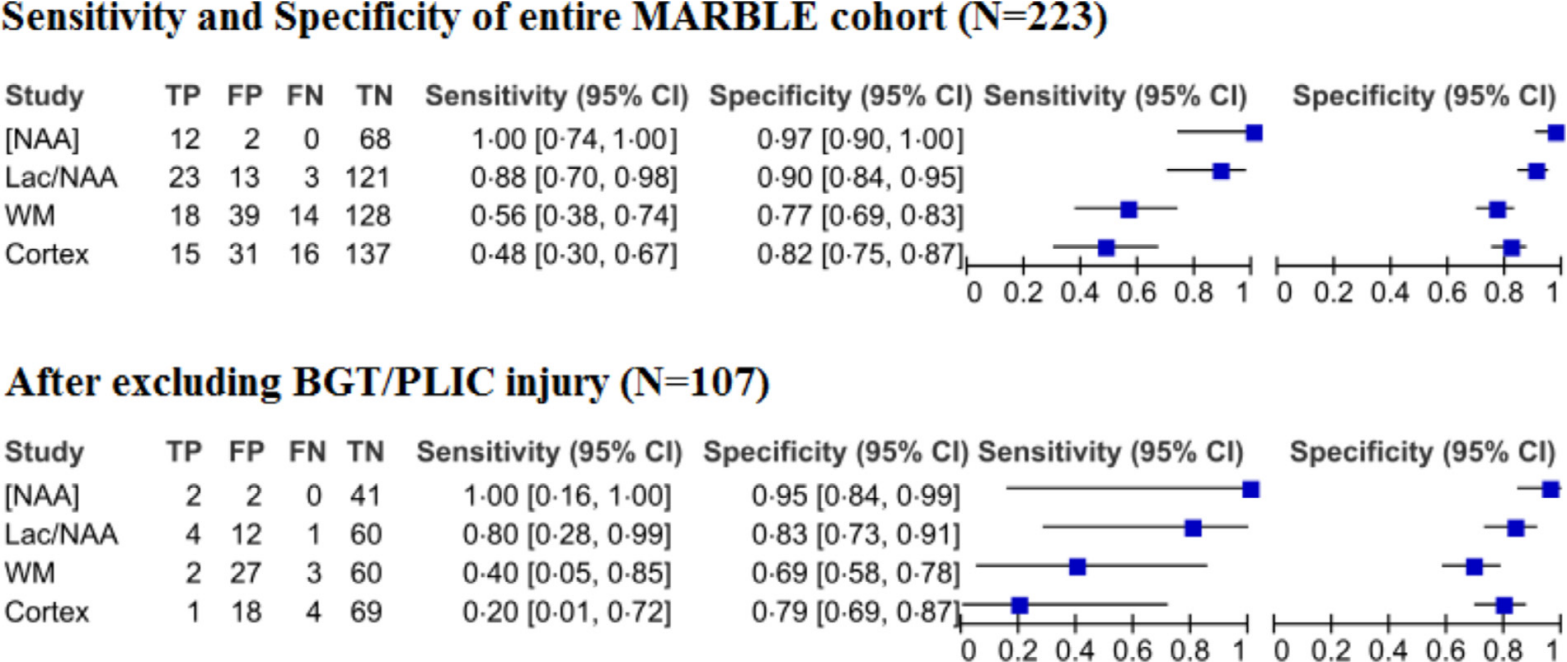
Forest plot demonstrating the sensitivity and specificity of thalamic [NAA], Lactate/NAA, NAA/Choline, NAA/Creatine, WM, and the cortex before and after excluding infants with BGT/PLIC injury.

**Fig. 3:**
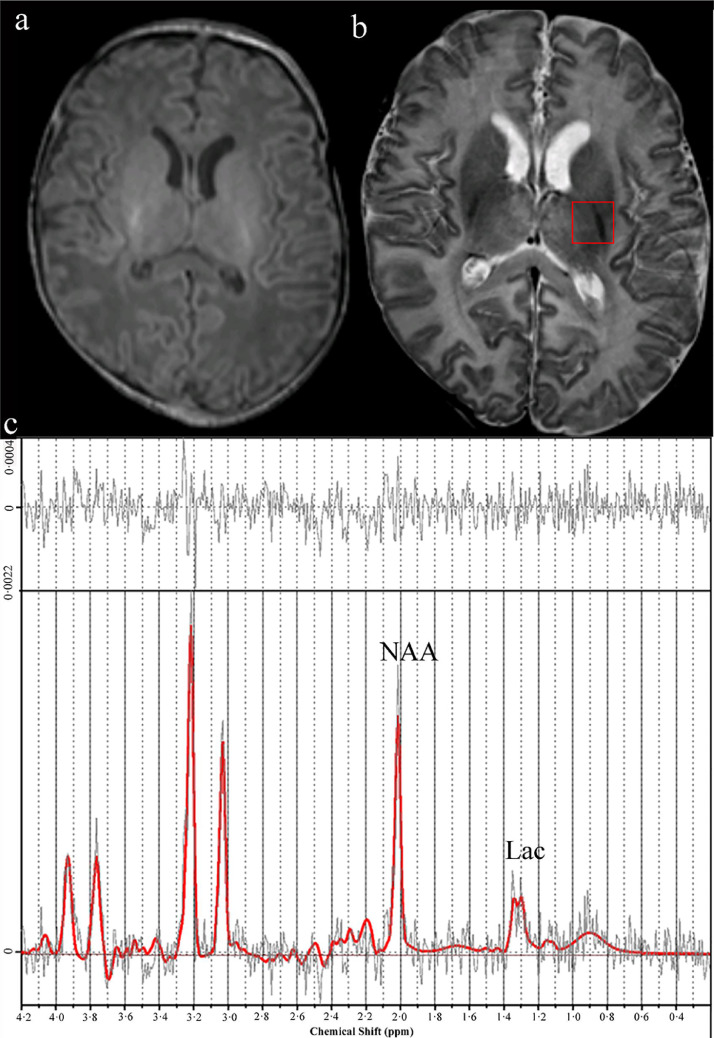
(a) Axial T_1_-weighted MR image showing normal basal ganglia and thalamic area and normal myelination in the posterior limb of internal capsule (b) Axial T_2_- weighted MR image showing injury in the frontal and occipital WM (arrowheads). The region of interest for the MR spectroscopy was in the left thalami (red rectangle). (c) Proton MR spectrum analysed by LC Model; [NAA] 4.65 mmol/kg/wet weight (low), Lac/tNAA 0.41, tNAA/Cr 1.19, tNAA/Cho 0.86. The infant had severe adverse outcome and cerebral palsy at two years.

WM and cortical injury scores on conventional MRI on their own had poor sensitivity (WM: 0.40 (95% CI 0.05–0.85), cortex: 0.20 (95% CI 0.01–0.72) and good specificity (WM: 0.69 (95% CI 0.58–0.79), cortex: 0.79 (95% CI 0.69–0.87) when predicting adverse outcome (Fig. 2, 3 and Table 2).

## 4 Discussion

In this secondary analysis of a large multicentre prospective study, we report that thalamic MRS abnormalities still occur in subcortical or periventricular WMI, even in the absence of MR visible injury to the deep brain nuclei. Thalamic [NAA] and Lactate/NAA were shown to be significantly associated with WMI. However, the prognostic accuracy of thalamic [NAA] (sensitivity 100% and specificity 95%) was higher than that of thalamic Lactate/NAA (sensitivity 80%, specificity 82%) in babies with WM and cortical injury. While some recent studies have presented Lactate/NAA as an accurate predictor of outcome and disability following neonatal asphyxia [17,18], our analysis suggests that thalamic [NAA] is a more robust surrogate marker of outcome in a mixed injury cohort.

WMI is common in preterm infants, but there is little evidence that WMI on neonatal MRI predicts later outcome [19]. In term babies, WMI has traditionally been associated with chronic sub-acute hypoxia and therefore, not involving areas with high metabolic activity like the BGT. However, if a prolonged and sustained hypoxic-ischaemic insult occurs, a global brain injury with a lack of grey matter/WM differentiation has been observed [20]. An investigation from the pre-cooling era described a significant association between BGT lesions and severe/moderate WMI in term infants with NE. In this study, an overt infarction was found in 14% of the patients who presented with encephalopathy after a sentinel event [21]. Previous data on 84 encephalopathic babies with watershed predominant injury pattern admitted to our neonatal unit showed some degree of BGT injury in 10% of the cases [22]. These findings suggest that perinatal WMI in term infants can have an impact on thalamocortical connectivity, thus leading to secondary degeneration of central grey matter [22]. However, in such cases, conventional MRI may not be sensitive enough to visualise the extent of injury occurring. Therefore, it is possible that these studies have been under-reporting thalamic injury, particularly in infants with mild hypoxic ischaemia.

Preclinical evidence, such as the work by Williams and Gunn, suggests that thalamic injury may occur even after brief episodes of ischaemia and in cases with mild hypoxic-ischaemic insults, whereas the hippocampus and parasagittal cortex are invariably involved [2]. A study by Stadlin et al. observed both BGT and WMI in an animal model of mild hypoxic ischaemia [23]. These preclinical findings contradict the traditional assumptions regarding BGT and WMI patterns by proposing the existence of BGT injury in cases other than acute or profound near-total hypoxia. Despite this exhibition of concurring BGT and WMI in animal models, the corresponding clinical evidence is lacking.

A potential obstacle to the use of thalamic MRS is that without an acute hypoxic event, the prognostic accuracy may be expected to be lower due to an assumed lack of BGT injury. In our study, only 15% of patients had recorded sentinel events, which would have ordinarily suggested a lack of thalamic injury within the cohort and therefore, a diminished predictive ability of thalamic MRS. Despite this, thalamic [NAA] (AUC:0.99, sensitivity:100%, specificity:95%) still allowed for early and accurate predictions of neurological outcome in our study.

Our secondary analysis also revealed that along with accurately predicting adverse outcomes, thalamic [NAA] was significantly associated with cognitive, language, and motor composite scores (Bayley-III) at two years in a subgroup of patients with mixed WM and cortical injury. In comparison, MRI grading of WM and cortical injury demonstrated relatively low sensitivity (40%; 20%). This underscores both the prognostic potential of MRS and its ability to compliment current MRI methods. Therefore, [NAA], having exhibited high prognostic accuracy irrespective of the underlying cause or patterns of brain injury, has potential as an effective surrogate outcome measure in early phase neuroprotection trials of neonatal encephalopathy.

While the absolute quantification of [NAA], as described in our study, could be considered the gold standard for multi-centre studies using different MR scanners (Siemens, GE and Phillips), there are several challenges in this approach. Firstly, the same protocol, parameters and post-processing methods need to be used to replicate the similar [NAA] levels. Any variation in protocol and post-processing methods may yield different [NAA] levels. Our sequences were specifically developed as cross-platform for multi-centre studies, although further optimisation for the specific scanner type may be possible. However, such vendor specific or proprietary sequences limit the comparisons of [NAA] levels across different scanners. Secondly, the acquisition time (25 min) for absolute quantification is longer than peak area ratio measurements and requires a clinical research agreement with the vendors. However, the information obtained from the MARBLE study has now enabled us to shorten the [NAA] acquisition time to 12 min, without the need for a clinical research agreement. This protocol has been successfully used as part of the recently completed HELIX (Hypothermia for Encephalopathy in Low and Middle-income countries, NCT02387385) trial, which has recruited 408 encephalopathic babies from India, Sri Lanka and Bangladesh [24], as well as in the ongoing COMET (Cooling in Mild Encephalopathy, NCT03409770) trial in the UK. Finally, careful and meticulous preparation of the baby so that the baby sleeps during the MR scan, is essential to avoid movement artefacts. We carefully adjusted feeding times, minimised noise and light, used light oral sedation, and a vacuum mattress during the MR scan. All scans were performed by MR-trained doctors and nurses. Movement artefacts and image degradation are common in routine clinical settings where a junior doctor or a clinical nurse, without specific MR-training, monitors the baby. Hence, absolute quantification may be difficult in such settings.

A main limitation of this study is its primary focus on thalamic MRS biomarker analysis, thus neglecting other potential highly active metabolic regions, such as the hippocampus, which has also been shown to sustain injury following acute HIE [20,25]. By failing to examine such regions we may be overlooking further MRS prognostic data. However, MRS in such areas would be more difficult considering both its size and location. Therefore, future work could explore other highly metabolic regions such as the somatosensory cortex or putamen [26]. Secondly, only a subset of the patients recruited underwent absolute quantification of thalamic [NAA], thus limiting the comparisons between the diagnostic accuracy (AUC) of NAA and Lactate/NAA. Thirdly, only five patients had adverse outcome. This is due to the inclusion of infants with only isolated WM or mixed WM/cortical injury, and not overt basal ganglia injury. The wide CIs of the sensitivity of [NAA] reflects this low event rate. The accuracy of [NAA] as a biomarker of isolated WMI needs to be evaluated further in large prospective studies. Several such studies, including the HELIX trial, are currently ongoing. Nevertheless, routine use of MRS in infants with isolated WMI may help to appropriately counsel the patients’ family. The implications of isolated WMI on later cognitive and behavioural outcomes during childhood remain unclear, hence, long-term follow-up studies are required to investigate this further. Fourthly, the age at the time of MRI varied significantly (4–14 days of age). However, in the MARBLE study, we did not find any relationship between postnatal age and the prognostic accuracy of [NAA] as long as the scans were performed within 4–14 days of age [11]. Finally, our study included only infants who had undergone therapeutic hypothermia. Therefore, the generalisability of these findings to neonates with WMI who do not undergo therapeutic hypothermia still needs to be evaluated. The strengths of this study include the harmonisation of the MRS across all centres, central and masked MRI and MRS analysis, and the sample size of the patient cohort. Also, as the MRI/MRS data was collected using scanners from three different vendors (Philips, Siemens, and GE Healthcare), this serves as a model of the true diversity of the clinical setting, further emphasising the predictive ability of [NAA].

Introducing MRS biomarkers into clinical practice when treating NE provides valuable information regarding metabolic perturbation. This information may compliment MR imaging data and contribute to earlier and more accurate predictions of neurological outcome following therapeutic hypothermia. Furthermore, the widespread availability of proton MRS in clinical settings in combination with standardised MR protocols would allow for further clinical trials to use MRS surrogate biomarkers in multi-centre studies.

In summary, this study highlights the clinical applicability of thalamic [NAA] as a more sensitive marker of injury in infants with NE compared to conventional MRI and other MRS biomarkers. The prognostic accuracy of thalamic [NAA] was shown to be independent of the pattern of brain injury on conventional MR imaging, considering the exclusion of infants with BGT/PLIC injury. Normal thalamic [NAA] in term infants with isolated WMI suggests a good outcome at two years of age, and therefore, may help to avoid unnecessary parental anxiety.

## Author contributions

PI, PM, ST, and PB had full access to the study data and take responsibility for the accuracy of the data analysis. ST (Chief Investigator) was responsible for conceptualization, funding aquisition, supervision, writing- review and editing. PI and PM wrote the original draft of the manuscript. PB performed formal statistical analysis. PJL and ST were responsible for the formal analysis of the magnetic resonance biomarkers. VO, JM and SP assisted in data curation and project administration. PM and RS were responsible for formal analysis of the neurodevelopmental outcome assessments. SS was responsible for formal analysis of neurodevelopmental outcome assessments and writing- review and editing. All authors critically revised the intellectual content of the manuscript.

## Declaration of Competing Interest

We declare no competing interests.

## Appendix Supplementary materials

Image, application 1
