# Supplementary material for: White matter injury after neonatal encephalopathy is associated with thalamic metabolite perturbations
Source: eBioMedicine. 2020 Feb 12;52:102663. doi: 10.1016/j.ebiom.2020.102663 (PMC7016374; doi:10.1016/j.ebiom.2020.102663)
Supplement: Supplementary file 1 [file mmc1.docx]

**Supplementary Materials**

**Supplementary Table 1.** Results of binary logistic regression showing association of MRS biomarkers with WM MRI scores (1 vs 2-3) unadjusted and adjusted for gestational age and age at time of MRI.

| **MRS Biomarker** | **Unadjusted** | | | **Adjusted** | | |
| --- | --- | --- | --- | --- | --- | --- |
|  | OR | 95% CI | *P* value | OR | 95% CI | *P* value |
| [NAA] | 0·40 | 0·19-0·85 | 0·02 | 0·40 | 0·18-0·93 | 0·033 |
| Lactate/NAA (*) | 3·40 | 1·56-7·44 | 0·002 | 3·37 | 1·45-7·82 | 0·005 |
| NAA/Choline | 0·09 | 0·01-2·34 | 0·15 | 0·214 | 0·01-7·31 | 0·39 |
| NAA/Creatine | 0·30 | 0·04-2·40 | 0·29 | 0·394 | 0·05-3·28 | 0·39 |

(*) *Odds ratio for Lactate/NAA ratio given for a 0*·*1-unit increase*

**Supplementary Table 2.** Results of the unadjusted binary logistic regression showing the relationship of MRS biomarkers with cortical MRI scores (0-1 vs 2-3).

| **MRS Biomarker** | **Cortical** | | |
| --- | --- | --- | --- |
|  | OR | 95% CI | *P* value |
| [NAA] | 1·03 | 0·13-8·14 | 0·98 |
| Lactate/NAA (*) | 1·68 | 0·53-5·33 | 0·38 |
| NAA/Choline | 7·48 | 0·01-5775·92 | 0·55 |
| NAA/Creatine | 25·55 | 0·26-2530·71 | 0·17 |

(*) *Odds ratio for Lactate/NAA ratio given for a 0*·*1-unit increase*

**Supplementary Table 3.** Results of the unadjusted binary logistic regression showing the association of MRS biomarkers with adverse outcome (yes/no) at two years.

| **MRS Biomarker** | **Outcome** | | |
| --- | --- | --- | --- |
|  | OR | 95% CI | *P* value |
| [NAA] | 0·06 | 0·004-0·88 | 0·04 |
| Lactate/NAA (*) | 5·97 | 1·86-19·14 | 0·003 |
| NAA/Choline | 0·87 | 0·002-327·17 | 0·96 |
| NAA/Creatine | 0·26 | 0·004-17·08 | 0·52 |

(*) *Odds ratio for Lactate/NAA ratio given for a 0*·*1-unit increase*

**Supplementary Table 4.** Clinical characteristics of patients with thalamic [NAA] quantification and Lactate/NAA quantification.

|  | Thalamic [NAA] quantification (N=49) | Lactate/NAA peak-area ratio (N=90) |
| --- | --- | --- |
| Gestational age (weeks) | 39·7 (1·79) | 39·6 (1·63) |
| Birth weight (kg) | 3·37 (0·55) | 3·37 (0·55) |
| Cord pH | 6·9 (0·19) | 6·9 (0·17) |
| Apgar 5 min | 4 [3] | 4 [3] |
| Seizures <6 hours | 23 (47%) | 41 (45·6%) |
| Intrapartum events  Ruptured uterus  Cord prolapse  Shoulder dystocia  Obstructed labour | 6 (12·2%)  1 (2%)  0 (0%)  2 (4·1%)  3 (6·%) | 12 (13·3%)  3 (3·3%)  0 (0%)  6 (6·7%)  3 (3·3%) |
| HIE Stage | | |
| Mild | 9 (18·4%) | 16 (17·8%) |
| Moderate | 38 (77·6%) | 66 (73·3%) |
| Severe | 2 (4·1%) | 7 (7·8%) |
| Age at MRI (days) | 7·65 (3·68) | 8·32 (4·07) |
| Adverse Outcome at 2 years | 2 (4·1%) | 5 (5·6%) |
| Positive blood culture | 2 (4·1%) | 2 (2·2%) |
| Hypoglycaemia | 11 (22·4%) | 19 (21·1%) |
| Cerebral palsy | 3 (6·1%) | 7 (7·8%) |
| Prolonged Rupture of Membrane | 9 (18·4%) | 19 (21·1%) |
| Reduced foetal movements | 10 (20·4%) | 13 (14·4%) |
| Abnormal cardiotocogram  Bradycardia  Late decelerations  Other abnormality  Variable decelerations  Sinusoidal | 41 (83·6%)  8 (16·3%)  5 (10·2%)  9 (18·4%)  3 (6·1%)  1 (2%) | 78 (86·7%)  20 (22·2%)  8 (8·9%)  15 (16·7%)  5 (5·6%)  2 (2·2%) |
| Antepartum haemorrhage | 1 (2%) | 6 (6·7%) |
| Meconium stained liquor | 24 (49%) | 35 (38·9%) |
| Emergency LSCS | 23 (46·9%) | 40 (44·4%) |
| Resuscitation  Bag and Mask/T Piece  Intubation  Cardiac Massage  Fluids  Drugs | 47 (95·9%)  16 (32·7%)  13 (26·5%)  10 (20·4%)  2 (4·1%)  1 (2%) | 82 (91·1%)  27 (30%)  25 (27·8%)  19 (21·1%)  5 (5·6%)  1 (1·1%) |
